# The Effect of Significant Exercise Modalities, Gender and Age on 9 Markers (Indicators) in NHISS Registered ACL Patients for Designing Exercise Intervention Program

**Published:** 2020-05

**Authors:** Hyunseok JEE, Sae Yong LEE

**Affiliations:** 1. Frontier Research Institute of Convergence Sports Science (FRICSS), Yonsei University, 50, Yonsei-ro, Seodaemun-gu, Seoul, 03722, Republic of Korea; 2. School of Kinesiology, Yeungnam University, 280 Daehak-ro, Gyeongsan, Gyeongbuk 38541, Republic of Korea; 3. Integrated Sports Science Research Laboratory (ISSRL), Yonsei University, 50, Yonsei-ro, Seodaemun-gu, Seoul 03722,Republic of Korea; 4. Department of Physical Education, College of Sciences in Education, Yonsei University, 50, Yonsei-ro, Seodaemun-gu, Seoul 03722, Republic of Korea

**Keywords:** Health insurance service database, Anterior cruciate ligament, Knee joint, Exercise types, Biomarker

## Abstract

**Background:**

Knee disease is prevalent in the post middle-aged and associated with lower quality of life. Knee disease (i.e., anterior cruciate ligament, ACL) related injury preventive program should be supported. We examined the significant effect of different age, gender, and exercise modalities on measureable nine dependent markers in National Health Insurance Sharing Service database (NHISS DB) registered ACL patients using big data analysis.

**Methods:**

The 1755 ACL patients from 514,866 in NHISS DB have been randomly selected by retrospective cohort study using big data from 2002 to 2013. Six independent and 9 dependent variables were used for analyzing patients with ACL injuries by T-test and Two-way analysis of variance (ANOVA).

**Results:**

Mean (SD) (men vs. women) of BMI, high blood pressure (BP), serum glutamic oxaloacetic transaminase (SGOT), and total cholesterol were 24.38±2.72 vs. 24.86±3.12 (*P*<0.01, 95% C.I., −0.763 ∼ −0.194), 126.64±14.70 vs. 125.02±16.62 (*P*<0.05, 95% C.I., 0.104 ∼ 3.151), 27.63±12.18 vs. 24.27±8.48 (*P*<0.01, 95% C.I., 2.393 ∼ 4.331), 197.77±37.60 vs. 205.72±36.72 (*P*<0.01, 95% C.I., −11.533 ∼ −4.378), respectively. Age and the frequency of 20 min severe exercise per week (Move20_Freq) intensive exercise had a significant association with BMI (*P*<0.05). Gender and Move20_Freq had a significant association with BP (*P*<0.05).

**Conclusion:**

Age-dependent Move20_Freq is associated with BMI in ACL patients. Women with ACL have higher BMI and cholesterol levels than men. These gender-specific differences can be relieved by exercise.

## Introduction

According to the annual statistic report of national health insurance in 2015, about 5.1% of Korean population, who visited hospitals for knee joint diseases, had paid about 10,000 USD and spent about an average of 29.1 days per person in the hospital (http://www.kostat.go.kr/). The patients with knee joint diseases have a wide age range, from 25 yr (exposed to initial pathogenesis of knee diseases) to >55 yr (intensive pathogenesis of knee diseases). Knee diseases in patients above 50 yr old are strongly associated with physical inactivity, which contributes to unhealthy longevity and has a major impact on quality of life (QoL) in later stages (1).

Anterior cruciate ligament (ACL) injuries have often been observed during sudden decelerating weight-bearing motions, such as landing, leading to an excessive contraction force of the quadriceps muscles at a shallow knee flexion angle, rupturing the ACL (2–4). An ACL injury treatment program should be seriously considered since ACL injury is one of the main causes of knee osteoarthritis (OA) (5,6). Hence, an ACL-related injury preventive program should be supported, since incapability of complete recovery from ACL injury usually results in worsening of the pathogenesis (7). By providing fundamental information on the association of physiological markers such as body mass index (BMI), blood pressure (BP), and waist circumference (Waist), associated with OA (8–11), this study can provide the basis for an effective knee injury preventive program. The role of different modalities of exercise and specific markers in ACL pathogenesis and cure is not clear. More studies are required to decide suitable intensity, duration, and exercise modality to design the ideal knee injury preventive program.

Currently, Korean medical insurance system is unilateral and all the information (e.g., out- or/and in-patients, medical treatment) is recorded, well organized, and stored in an electronically digitized format, and can provide uniform results flexibly designed as intended. Big data indicates large-scaled dataset that is difficult to collect, access, store, manage, and analyze by using usual database management system (12). Characteristic analysis of the knee joint injury is available from the National Health Insurance Sharing Service (NHISS) (https://nhiss.nhis.or.kr/) that mandates the enrollment of all Korean patients.

The knee joint related injuries analyzed by using big data set have been rarely performed, and the relationship between knee damage and the physiological markers is not clearly understood. Importantly, there has been no NHISS DB based study reported for developing exercise intervention program. Concretely speaking, scientific results based reasonable age, gender, exercise modalities (i.e., types, time, intensity, frequency) for the therapeutic and prognostic effects on the post middle aged ACL patients have not been revealed. Furthermore, there has been lack of information on how those factors are significantly associated with other indicators, which can be also useful information for the prevention of ACL symptoms for the public health. We thus hypothesized that: 1. physiologically related markers included in NHISS such as BMI, BP, serum glutamic oxaloacetic transaminase (SGOT), serum glutamic-pyruvic transaminase (SGPT), total cholesterol (TOT_CHOLE), etc. show dissimilar levels in different genders. 2. Gender differences, age, and exercise modalities (independent parameters) affect various indicators such as BMI, BP, SGOT, SGPT, TOT_CHOLE etc. (dependent parameters).

In this study, we aimed to examine these hypotheses to know the significant effect of different age, gender, and exercise modalities on measureable markers in NHISS registered ACL patients using big data analysis. This study may provide evidence-based scientific information that could contribute to developing a preventive program for patients with ACL injuries.

## Materials and Methods

### Data source, subjects’ population, and study procedure

**Fig. 1:**
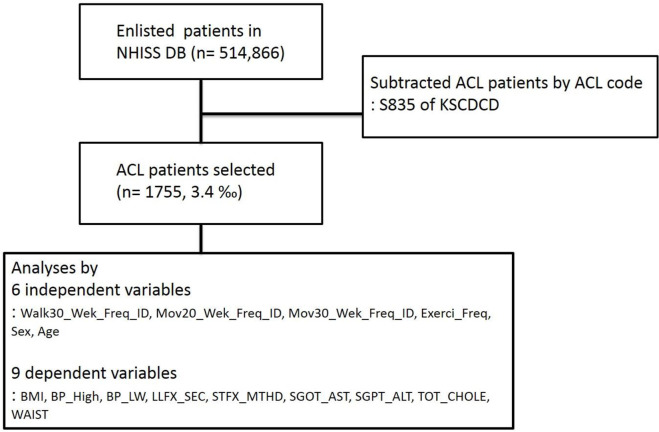
Anterior cruciate ligament (ACL) patients subtracting process Anterior cruciate ligament (ACL) patients (n= 1755) were selected from the National Health Insurance Sharing Service Data Base (NHISS DB). ACL, anterior cruciate ligament; n, number; KSCDCD, Korean standard classification of disease and causes of death; Exerci_Freq, the number of dates on exercise; Walk30_Wek, the date number of over 30 min walking exercise in a week; Move20_Wek, the number of dates on performing 20 min intensive exercise in a week; Move30_Wek, the number of dates on performing 30 min moderate exercise in a week; BMI, body mass index; BP_High, maximal systolic blood pressure; BP_LW, lowest diastolic blood pressure; LLFX, lower extremity function assessing times taken by going 3 m away and back (only for 66-year-olds); STFX, balance test only for 66-year-olds by measuring time of standing up with/without eyes closed; SGOT_AST, serum glutamic oxaloacetic transaminase aspartate aminotransferase; SGPT_ALT, serum glutamate-pyruvate transaminase alanine aminotransferase; TOT_CHOLE, total cholesterol.

All Korean citizens have to register mandatorily with NHISS for their health checkup every 2 years (which indicates there is unnecessary to recruit the medical record of the completely Korean population because it had been automatically recorded). In total, 10% (about 510,000) of unidentified Korean citizens with medical records in NHISS DB were randomly selected from around 5 million subjects within the age group of 40 to 79 yr in Dec 2002. The 510,000 subjects’ records have been tracked for 12 years i.e. from 2002 to 2013 (which provided from NHISS as the dataset collected during the 12 years from 2002 to 2013) and ACL patients were selected (n= 1755, ACL code: S835 by the Korean Standard Classification of Disease and Causes of Death (KSCDCD, http://kssc.kostat.go.kr/ksscNew_web/index.jsp). All subjects were selected according to 15 parameters (6 independent variables and 9 dependent variables described in Fig. 1) using the SAS software; among KSCDCD registered whole disease codes, ACL patients groups were extracted by using S835 commanding code (e.g., if sick name = ‘S835’ then subtracted the ACL patients and locate the subtracted patients to the appointed location). (Fig. 1). There is no missing data in the NHISS DB; however, non-responded answers by the subjects were not included in the analyses. KSCDCD indicates that S835 defines sprain and strain involving anterior or posterior cruciate ligament of knee. All data were obtained by using actual measurement values or medical questionnaires (e.g., the answer to exercise frequency 1 indicates one-day exercise per week). Significant parameters were calculated and output by using statistical methods described in the *Statistical analysis*.

The study was approved by the institutional review board at Yonsei University (1040917-201603-HRBR-152-01E).

### Dependent and independent parameters

Each of the five age groups (40 to 49 yr, 50 to 59 yr, 60 to 69 yr, 70 to 79 yr, and 80 to 89 yr), gender, and four different modalities of exercise (Exerci_Freq, frequency with moderate intensity of exercise per week; Move20_Freq, frequency of 20 min severe exercise per week; Move30_Freq, frequency of 30 min moderate intensity exercise per week; Walk30_Wek, frequency of 30 min walking per week) are categorized as independent variables. As dependent variables, BMI, (kg/m^2^), high BP (BP_High mmHg), low BP (BP_LW mmHg), lower limb function test for 66-year-old subjects (LLFX, time in seconds from standing up from a chair, walking 3 m and returning to the same chair), balance test for 66-year-old subjects (STFX, standing time in seconds on one leg with eyes closed or open), SGOT and aspartate aminotransferase (SGOT_AST U/L) levels, SGPT and alanine aminotransaminase (SGPT_ALT U/L) levels, TOT_CHOLE, (mg/dL) levels, and Waist (cm) are selected.

### Statistical analysis

All data are presented as the mean (S.D.). The normality was confirmed by using Shapiro-wilk test. Homogeneity of variance was checked by using Levene’s test and *P*-values were then provided via using t-test and two-way analysis of variance (ANOVA). Confounding factors in the two-way ANOVA were adjusted. Sex were regarded as independent variables. The interaction between each modalities of exercise type, each of the five stratified age groups, and each gender as independent variables was verified for their effect on the dependent variables using two-way ANOVA. SAS version 9.4 (SAS Institute, Cary, NC, USA) was used for all statistical analyses. A value of *P*<0.05 was considered to indicate a statistically significant difference in all analyses.

## Results

### Study populations

A randomly selected population of 514,866 subjects is representative of almost 5 million Korean citizens aged over 40 yr (Table 1). Overall, 1755 subjects were selected, who met the criteria defined by KSCDCD code for ACL injuries. The average age of patients with an ACL injury is 52.6 yr, computed from the huge age group ranging from 40 to 80 yr.

**Table 1:** Characteristics of NHISS DB-derived ACL population

***Parameters***	***Values***
Total population	514,866
Age (yr)	57.43 ± 10.10
Following-up years (yrs)	2002 to 2013
Subtracted ACL patients by S835 in 2002	1,755

KSCDCR-derived code S835-used ACL patients (n=1755) were subtracted from NHISS DB. NHISS DB, National health insurance sharing service data base; KSCDCR, Korean standard classification of disease and causes of death; yrs, years; ACL, anterior cruciate ligament.

### The characteristics of ACL patients according to gender difference

Gender differences reflect the following parameters.

In ACL patients from 2002 to 2013, BP_High (*P*<0.05), BP_LW *(P*<0.001), SGOT_AST (*P*<0.001), SGPT_ALT (*P*<0.001), and Waist of men are significantly higher than in women. LLFX, STFX in men are seemingly higher than in women; however, there is no statistically significant difference. In women, BMI and TOT_CHOLE are higher than in men (*P*<0.001).

### Independent variables that are beneficial to BMI

**Fig. 2:**
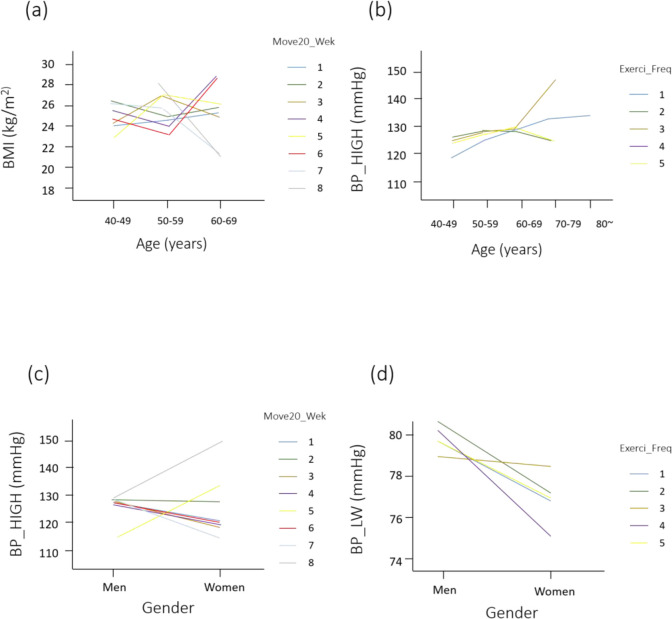
The change of specific indicators according to exercise types, age, and gender. (a), (b), (c), and (d) show the changes of each physiological indicator such as BMI (kg/m^2^) (a), BP_High (mmHg) (b, c), and BP_LW (mmHg) (d) according to different age, gender on various exercise modalities. BMI, body mass index; BP_High, high blood pressure; BP_LW, low blood pressure; Mov20_Wek, Frequency of 20 min with severe exercise per week; Exerci_Freq, Frequency with moderate intensity of exercise per week. 1 (40 to 49 yrs), 2 (50 to 59 yrs), 3 (60 to 69 yrs), 4 (70 to 79 yrs), and 5 (80 yrs) in Age and 1 (Men) and 2 (Women) in gender. 1 (not at all), 2 (one time per week), 3 (two times per week), 4 (three times per week), 5 (four times per week), 6 (five times per week), 7 (six times per week), and 8 (exercise on everyday basis) in each Exercise type

Two-way ANOVA analysis indicates that age difference (*P*<0.001) or the interaction between age and Move20_Freq (*P*<0.05) significantly affects the differences in BMI (Table 2). There is no regular pattern as shown in Fig. 2a. However, a noticeable trend from 40 to 59 yr shows that higher the frequency of Move20_Freq exercise in each age group more is the decrease in BMI value. Regarding the BMI-regulating modality of exercise type, Move20_wek can be recommended over other types of exercise according to these results (Table 2) (Fig. 2a).

**Table 2:** 2 way ANOVA for parameter comparisons between 9 dependent and 6 independent variables

***Dependent variables***	***Independent variables (P-values)***			***Interaction (P-values)***	***Exercise type***
			
	Gender	Exercise	Age		
BMI (kg/m^2^)				√ (0.040, Mov20_Freq&Age)	Mov20_Wek
BMI (kg/m^2^)	√ (0.007)		√ (0.000)	√ (0.000, Gender&Age)	N/A
BP_High (mmHg)			√ (0.000)		Exerci_Freq
BP_High (mmHg)			√ (0.005)		Mov20_Freq
BP_High (mmHg)				√ (0.039, Mov20_Freq&Gender)	Mov20_Freq
BP_High (mmHg)			√ (0.000)	√ (0.018, Mov30_Freq&Age)	Mov30_Freq
BP_High (mmHg)			√ (0.000)	√ (0.000, Gender&Age)	N/A
BP_High (mmHg)			√ (0.000)		Walk30_Wek
BP_LW (mmHg)	√ (0.001)				Exerci_Freq
BP_LW (mmHg)			√ (0.015)		Mov30_Freq
BP_LW (mmHg)			√ (0.042)	√ (0.000, Gender&Age)	N/A
BP_LW (mmHg)	√ (0.039)			√ (0.017, Walk30_Wek&Gender)	Walk30_Wek
LLFX (sec)	√ (0.026)	√ (0.038)		√ (0.049, Walk30_Wek&Sex)	Walk30_Wek
SGOT_AST (U/L)	√ (0.000)				Exerci_Freq
SGOT_AST (U/L)			√ (0.023)		Mov30_Freq
SGOT_AST (U/L)			√ (0.044)	√ (0.039, Gender&Age)	N/A
SGOT_AST (U/L)	√ (0.03)				Walk30_Wek
SGPT_ALT (U/L)	√ (0.03)		√ (0.006)	√ (0.005, Gender&Age)	N/A
SGPT_ALT (U/L)	√ (0.005)				Walk30_Wek
TOT_CHOLE (mg/dL)	√ (0.01)				Exerci_Freq
TOT_CHOLE (mg/dL)	√ (0.000)		√ (0.008)	√ (0.000, Gender&Age)	N/A
TOT_CHOLE (mg/dL)	√ (0.009)				Walk30_Wek
Waist (cm)	√ (0.000)	√ (0.019)			Mov20_Freq
Waist (cm)			√ (0.005)		Mov30_Freq
Waist (cm)	√ (0.000)				Mov30_Freq
Waist (cm)	√ (0.000)		√ (0.000)	√ (0.001, Gender&Age)	N/A
Waist (cm)	√ (0.000)	√ (0.019)			Walk30_Wek

BMI, body mass index; BP high, the highest blood pressure; BP lw, the lowest blood pressure; LLFX, lower limb function test for only 66 year old ages (time from standing up from chair to go 3 m and sitting in the same chair after coming); STFX, balance test for only 66 year old ages by standing with one leg (1, with closed eyes; 2, with opened eyes); SGOT_AST, serum glutamic oxaloacetic transaminase aspartate aminotransferase; SGPT_ALT, serum glutamic pyruvic transaminase alanine aminotransaminase; TOT_CHOLE, total cholesterol; Mov20_Wek, Frequency of severe exercise for 20 min in a week; Exerci_Freq, Number of times for exercise in a week; Mov30_Wek, Frequency of intermediate level of exercise for 30 min in a week; N/A, non-applicable

### Different Age, Gender, and Exercise modality on BP, LLFX, and STFX

**Fig. 3:**
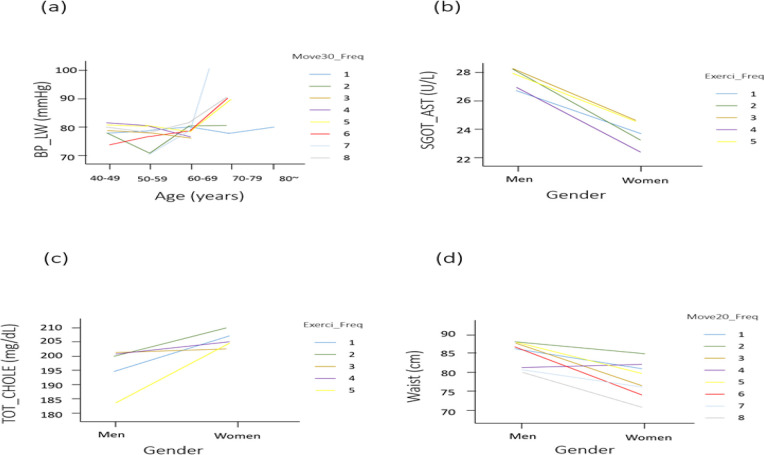
BP, SGOT, Cholesterol, and Waist level according to Move20, Move30, and Exerci_Freq in different ages and genders BP_LW according to different frequency of Move30_Freq with different age is shown in (a). (b) and (c) indicate SGOT and TOT_CHOLE in gender difference with exercise, respectively. Waist circumference changes by gender with different Move20_Freq is shown in (d). BP_LW, low blood pressure (mmHg); SGOT_AST (U/L), serum glutamic oxaloacetic transaminase aspartate aminotransferase; TOT_CHOLE, total cholesterol (mg/dL); Waist, waist circumference (cm); Move20_Freq, Frequency of 20 min severe exercise per week; Move30_Freq, Frequency of 30 min moderate intensity exercise per week; Exerci_Freq, Frequency with moderate intensity of exercise per week. 1 (40 to 49 yrs), 2 (50 to 59 yrs), 3 (60 to 69 yrs), 4 (70 to 79 yrs), and 5 (80 yrs) in age and 1 (Men) and 2 (Women) in gender. 1 (not at all), 2 (one time per week), 3 (two times per week), 4 (three times per week), 5(four times per week), 6 (five times per week), 7 (six times per week), and 8 (everyday exercise) in each Exercise type

BP_High showed an increasing pattern as age advances (Fig. 2b). Gender and Move20_Freq were significantly associated (*P*<0.05). Men who performed Move20_Freq had generally higher BP_High than women who performed the same test (Fig. 2). In the age-related assessment, a significant difference was observed in the interaction between age and Move30_Freq (*P*<0.05). Exercise frequency thus seems to affect the level of BP_High. Gender and Walk30_Wek were significantly associated with BP_LW (*P*<0.05), indicating that gender has different effects on BP_LW. However, it should be accompanied by exercise such as Walk30_Wek. Women seemingly have lower BP_LW. However, this difference was not statistically significant (Fig. 2d). Significant differences were observed in the interaction between gender and age (*P*<0.05). Age-dependent differences in BP_LW were not likely consistent (Fig. 3a). Walk30_Wek had significant association (*P*<0.05) with lower limb function (LLFX) than did other types of exercise. The balance test (STFX) had not shown any significant difference with any independent variable (Table 2).

### Gender, Age, and Exercise type difference affect SGOT_AST, TOT_CHOLE, and Waist

Significantly higher SGOT_AST were seen in men than in women (*P*<0.05) (Fig. 3b). Move30_Freq significantly affects SGOT_AST value (*P*<0.05). Unlike the exercise type, gender (*P*<0.05) or interactions between gender and age (*P*<0.01) affect SGPT_ALT. Women have higher TOT_CHOLE (*P*<0.05) (Fig. 3), however, any type of exercise modality does not significantly affect TOT_CHOLE. Men have higher value of Waist (*P*<0.001) than women and Move20_Freq lowered its values (*P*<0.05) (Fig. 3d) (Table 2).

## Discussion

We obtained 1755 ACL patients from NHISS DB clustering, the retrospective cohort of Koreans above 40 yr old. Regarding gender differences in physiological factors, the factors such as BP (*P*<0.05), SGOT (*P*<0.01), and SGPT (*P*<0.01) are higher in men, while BMI (*P*<0.05) and TOT_CHOLE (*P*<0.01) are higher in women. This study also revealed that specific exercise types such as Move20_Freq are associated with BMI (*P*<0.05), BP_High (*P*<0.01), and Waist (*P*<0.05). Move30_Freq has a significant impact on SGOT_AST value (*P*<0.05), whereas Walk30_Wek affects BP_LW (*P*<0.05), LLFX (*P*<0.05), and STFX (*P*<0.05).

### Gender differences in specific factors in elderly ACL patients

Irrespective of attempts made to repair ACL injury, even by performing biological graft replacement surgery, no proliferative and angiogenic response in damaged ACL has been observed, especially in the elderly (13). Vascular endothelial growth factor (VEGF), an essential factor in the repair process, is reported to decrease during the aging process possibly resulting in decreased angiogenesis leading to reduced capacity for repair in ACL injury in the elderly (14). This physiological malfunction at the molecular level can be responsible for incomplete repair, leading to the development of OA in 4% of cases following ACL damages. This post-traumatic OA is not caused by biomechanical but biochemical factors such as synovial fluid cytokine levels (15).

Studies explaining the correlation between SGOT_AST and SGPT_ALT are rare. We found only two previous studies (using the keywords such as SGOT_AST/SGPT_ALT, ACL/OA, or/and physiological marker, etc.) on knee OA treatment with nabumetone and oxaprozin, which caused elevation in SGPT_AST and SGOT_ALT (16,17). However, these studies revealed an elevation in SGPT_AST and SGOT_ALT in the post-medication period with nabumetone and oxaprozin without providing any detailed explanation on correlation or any possible mechanism.

In this study, we revealed the relationship between gender difference and quantity levels of dependent variables in ACL patients (Table 2). Interestingly, age is directly related to BP_High (Fig. 2b)

### Effective exercise types on ACL patients

This study can contribute to the development of an important method to manage risk factors affecting malignant chronic knee diseases such as OA post-ACL injury. Exercise as one of the interventions is suggested and this study provides the following interesting implications. Three (Move20_Freq, Move30_Freq, and Walk30_Wek) in NHISS significantly affect six (BMI, BP, Balance, SGOT, and Waist). It is worth considering why only Move20_Freq among many exercise types significantly affects only BMI and BP_High (Table 2) (*P*<0.05). Only Walk30_Wek affects LLFX (low limb function) and STFX (balance evaluation) (*P*<0.05). Move30_Freq and Move20_Freq affect SGOT_AST and Waist, respectively (*P*<0.05) (Table 2) (Fig. 3d). This suggests that non-identical time and different intensities of exercise can optimally and efficiently induce responses of the specific markers shown in this study. Results of Messier et al. in 2013 showed that widespread available designs of the intervention of exercise trials potentially brings positive clinical impact and these findings are consistent with our results (18). Each of the parameters recorded in ACL patients sourced from NHISS DB is an independent variable (e.g. different exercise modalities), specifically activated or inactivated, so that parameter specific target treatment by using different ranges of intensity, duration, and frequency of exercise treatment is possible. This study can be used for designing exercise-based treatment program for ACL patients.

### Advantage and disadvantages of big data analysis

Nationwide Inpatient Sample (NIS) in the US and National Health Insurance Research Database (NHIRD) has been founded by sampling a DB via medical treatment-derived hospital statements (19,20). Comparatively, all Korean citizens should be registered in the NHISS DB so that it is possible to trace their whole medical history (21). NHISS DB is undoubtedly better than the previous DB that had issues such as privacy concerns of individual information and a limited DB size making the researchers’ access difficult. However, NHISS DB provides computed raw DB derived results without those issues described above. The results made from the provided NHISS DB is well-constituted and sourced population (n=514,866) used in this study is enough sample size to generalize the results made in this study. Moreover, NHISS DB-used study provides qualitative results via cross-sectional DB, making large-scale sample subtraction possible.

### Limitation of our study

The questionnaires-responded patients with ACL might have a tendency with their subjective point views rather than their objective disease state based unbiased aspect even though large-scaled population DB survey possibly offsets these errors.

Large scale-based patients by patients longitudinally tracking study may be necessary for the next study to know the factors affecting the incidence of ACL.

## Conclusion

These newly found evidence-based results of study can help to standardize efficient exercise intervention programs for delaying, ameliorating, and preventing progression of knee damage caused by ACL injury, which eventually results in development of OA. The developing exercise program (controlling frequency, time, type, etc.) for knee diseases should be individualized to the target patients (e.g., specific age, gender, etc.) with significant markers of the parameters found from this large-scaled NHISS DB based analyses. It is essential to develop policies and support systems via using the beneficial information provided by this study since it will possibly contribute to not only in saving medical expenses but also in improving QoL of the post middle-aged people.

## Ethical considerations

Ethical issues (Including plagiarism, informed consent, misconduct, data fabrication and/or falsification, double publication and/or submission, redundancy, etc.) have been completely observed by the authors.
